# Stercoral Colitis: A Diagnostic Challenge and Therapeutic Approach in an Elderly Patient With Chronic Constipation

**DOI:** 10.7759/cureus.39179

**Published:** 2023-05-18

**Authors:** Hamad Ahmad, Hoore Jannat, Urooj Khan, Noaman Ahmad

**Affiliations:** 1 Internal Medicine, Westchester Medical Center, Valhalla, USA; 2 Internal Medicine, Khyber Medical College, Peshawar, PAK; 3 Internal Medicine, Khyber Medical University, Peshawar, PAK; 4 Internal Medicine, Huntsville Hospital, Huntsville, USA

**Keywords:** diagnostic challenge, chronic constipation, acute colonic inflammation, fecal impaction, stercoral colitis

## Abstract

Stercoral colitis, although rare, remains a significant cause of acute colonic inflammation. It is characterized by the presence of fecaloma leading to fecal impaction and subsequent mucosal injury, ultimately resulting in colonic wall inflammation. This condition primarily affects elderly patients with chronic constipation and is associated with significant morbidity and mortality if not recognized and managed promptly.

Given its rarity and varied presentation, stercoral colitis often poses a diagnostic challenge. The clinical manifestations can mimic other colonic pathologies, such as diverticulitis, ischemic colitis, and inflammatory bowel disease, further contributing to diagnostic dilemmas. However, an astute clinician, armed with a high index of suspicion and the aid of advanced imaging techniques, can establish the correct diagnosis and initiate timely management.

In this case report, we present a challenging case of stercoral colitis in an elderly patient with a history of chronic constipation. The aim of this report is to enhance awareness and understanding of this underdiagnosed condition among healthcare providers. Additionally, we discuss the clinical presentation, diagnostic workup, and therapeutic interventions employed to manage this formidable gastrointestinal entity.

## Introduction

Stercoral colitis primarily affects the sigmoid colon and rectum, where ulceration can occur if it is severe [1]. It is most frequently observed in patients with a history of chronic constipation, elderly individuals with dementia, bedbound or nursing home residents, and occasionally young patients with psychiatric conditions [2].

The pathogenesis of stercoral colitis involves the progression from chronic constipation to fecal impaction, resulting in distention and deformity of the colon, which leads to the formation of fecalomas (dehydrated fecal masses) [3]. These fecalomas become lodged within the colon and exert ischemic pressure on the colonic wall, leading to ulceration. If left untreated, focal ischemic necrosis may occur, potentially progressing to colonic perforation [3].

## Case presentation

**Figure 1:**
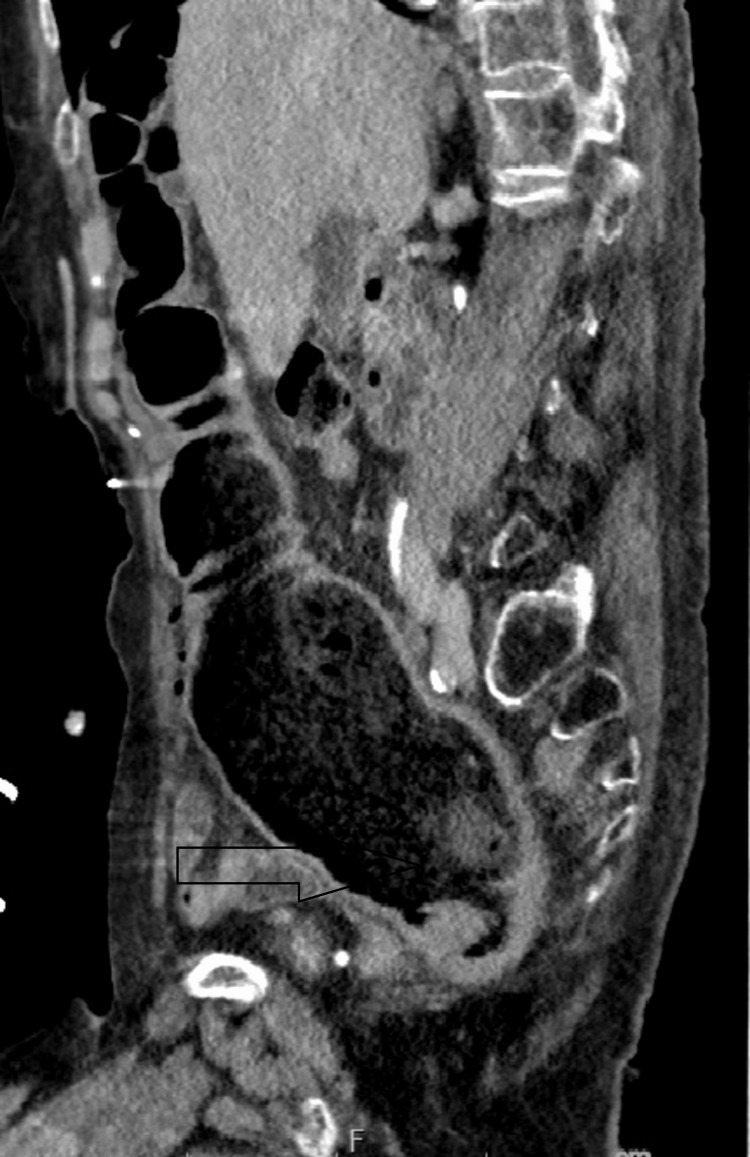
Computed tomography abdomen shows dilated inflamed thickened rectosigmoid

**Figure 2:**
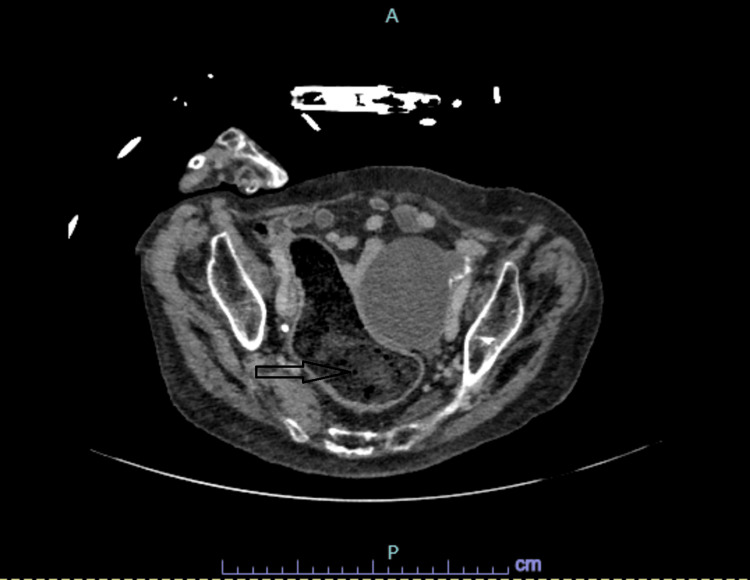
Computed tomography abdomen shows dilated inflamed thickened rectosigmoid

We present a complex case of an 83-year-old woman with a past medical history significant for anxiety, depression, meningioma, and Parkinson's disease. The patient was brought to the hospital after being found unresponsive at home. The initial assessment revealed a history of recent gastrointestinal symptoms, including diarrhea, emesis, and fever. Upon arrival at the hospital, on physical examination, the patient had altered mental status with signs of neurological impairment, including mild right-sided facial droop and a soft, non-distended, mildly tender abdomen. Computed tomography (CT) of the head revealed (stable) three extra-axial masses, localized in the right parietal lobe, left cerebellum, and retro-cerebellar cistern, consistent with known meningiomas. Additionally, severe atrophy and a chronic infarct in the left lentiform region were noted. Concurrently, a CT angiogram ruled out the presence of a large vessel occlusion suggestive of stroke. Blood work revealed leukocytosis (18,000 white blood cells); the rest of the routine labs, including urinalysis, did not show significant abnormalities. Of note, the patient was incidentally found to be positive for coronavirus disease 2019 (COVID-19) despite being asymptomatic. Given the negative neurologic work-up and new-onset altered mental status with fever, leukocytosis, and a mildly tender abdomen, the patient underwent CT abdomen and pelvis with contrast, which demonstrated severe stercoral proctocolitis, primarily affecting the sigmoid colon and rectum (Figures 1, 2). Moreover, the CT abdomen and pelvis also identified an 8.5 cm cystic mass in the pelvis.

To address the various medical concerns, the initial management involved administering levetiracetam for possible seizure activity and starting piperacillin or tazobactam for leukocytosis and fever given the findings of stercoral colitis on imaging to cover gram-negative organisms and anaerobes. Our patient did not show signs and symptoms of peritonitis and was therefore treated conservatively. The patient received a liberal bowel regimen and underwent manual disimpaction daily. Gastroenterology was consulted for consideration of endoscopic disimpaction, and they recommended continuing the current management with the addition of mineral oil enemas twice a day and polyethylene glycol 1 liter every 12 hours via nasogastric tubes, serial abdominal exams, and daily abdominal X-rays. They will consider endoscopic intervention if there is no resolution in stool burden in the next 48-72 hours. After receiving the appropriate treatment, the patient showed a remarkable improvement in her neurological status and became more alert and oriented. However, the patient continues to have a significant stool burden on the abdominal X-ray. The patient underwent flexible sigmoidoscopy, which showed thick, pasty stool in the sigmoid and descending colon with no hard stools or impaction. Fleet enemas were administered through the colonoscope channel to help soften and mobilize stools. Over the next 24 hours, the patient's mental status is completely back to baseline, with resolution of colonic and small bowel distention and subsequent resolution of colonic inflammation.

## Discussion

Stercoral colitis, being rare and variable in presentation, often poses a diagnostic challenge. The clinical manifestations can mimic other colonic pathologies. However, an astute clinician, armed with a high index of suspicion and the aid of advanced imaging techniques, can establish the correct diagnosis and initiate timely management.

Evaluation includes obtaining a detailed history of current presentation and comorbidities, a physical examination, blood work, and imaging. Physical exams and lab studies are generally nonspecific and, hence, can make it difficult to diagnose. Lab studies may show elevated leukocytes and acute phase reactants. It may cause elevated lactic acid and metabolic acidosis, especially if complications develop such as abscess formation or bowel perforation. Patients with high suspicion of complications should have an upright chest X-ray to evaluate for free air under the diaphragm and assess for bowel perforation [4]. CT abdomen and pelvis with contrast is the most sensitive and specific modality; findings include colonic distention, colonic wall thickening, diffuse wall edema, pressure necrosis, or ulceration [4]. The differential diagnoses for stercoral colitis include diverticulitis, large bowel obstruction, ulcerative colitis, infectious colitis, malignancy, bowel perforation, acute mesenteric ischemia, and intra-abdominal abscess [5].

Patients who do not exhibit signs of peritonitis can be managed conservatively without the need for surgery [2]. Non-operative approaches include manual disimpaction via the rectum or endoscopically guided disimpaction, along with the implementation of a bowel regimen and close monitoring during hospital admission [4]. Patients with sepsis or septic shock need appropriate resuscitation measures, including intravenous fluid administration and broad-spectrum antibiotics covering gram-negative and anaerobic organisms [1]. Operative management is reserved for patients who have perforation, extensive bowel involvement, or cases where conservative management has been unsuccessful [2]. The prognosis of stercoral colitis is closely tied to timely diagnosis and management. The most common serious complication of stercoral colitis is perforation, which carries a high risk of mortality. Other complications include sepsis, septic shock, ischemic colitis, and urinary retention caused by compression from a dilated bowel [6].

Investigation into the underlying cause of fecal impaction leading to stercoral colitis is essential. If chronic constipation contributes to the development of stercoral colitis, a bowel regimen should be initiated, and outpatient gastroenterology follow-up should be arranged to prevent disease recurrence. If other factors, such as neuropsychiatric disorders or opioid use, contributed to the condition, appropriate interventions involving social services, psychiatry, or pain management should be undertaken to optimize the patient's condition for safe discharge.

## Conclusions

In conclusion, stercoral colitis is a rare but significant cause of acute colonic inflammation, predominantly affecting elderly patients with chronic constipation. Early recognition and appropriate management are paramount to preventing potentially life-threatening complications. This case report serves to increase awareness among clinicians and highlight the diagnostic and therapeutic approaches necessary for optimal patient care.
